# Trends and emerging directions in HIV risk and prevention research in the Philippines: A systematic review of the literature

**DOI:** 10.1371/journal.pone.0207663

**Published:** 2018-12-05

**Authors:** Arjee Restar, Mary Nguyen, Kimberly Nguyen, Alexander Adia, Jennifer Nazareno, Emily Yoshioka, Laufred Hernandez, Don Operario

**Affiliations:** 1 Department of Behavioral and Social Sciences, Brown University School of Public Health, Providence, Rhode Island, United States of America; 2 Department of Behavioral Sciences, University of the Philippines, Manila, Philippines; University of South Florida, UNITED STATES

## Abstract

**Background:**

The Philippines is experiencing one of the fastest growing epidemics globally. Evidence-based public health policies are needed. To describe the public health literature on HIV risk groups and prevention approaches in the Philippines, we reviewed published empirical studies with HIV-related outcomes.

**Methods:**

Based on an a priori systematic review protocol, we searched PubMed, PsycINFO and CINAHL databases for quantitative studies conducted in the Philippines that reported on HIV risk groups factors and interventions to prevent HIV. The search included studies published as of April 2018.

**Results:**

We identified 755 records, screened 699 unique titles and abstracts, and conducted full text review of 122 full reports of which 51 articles met inclusion criteria. The majority were cross-sectional studies describing HIV and STI prevalence and risk factors in samples recruited from the Philippines. Four HIV prevention programs conducted in the Philippines were identified, all of which reported improvements on HIV knowledge, attitudes, and behaviors. Overall, female sex workers (FSWs) constituted the primary study population, and few studies reported data from men who have sex with men (MSM), people who inject drugs (PWIDs), and youth. No studies reported on transgender populations. Most studies were focused on examining condom use-related outcomes and STI history, few had biomarkers for HIV, and none addressed biomedical HIV prevention strategies.

**Conclusion:**

This review identifies an agenda for future HIV research that is needed to address the growing and shifting nature of the HIV epidemic in the Philippines.

## Introduction

After the first HIV case was identified in the Philippines in 1984, the country’s estimated HIV prevalence had remained low for over two decades [1]. According to the Joint United Nations Programme on HIV/AIDS (UNAIDS)’s surveillance reports, the Philippines’ progress towards reaching HIV/AIDS 90-90-90 treatment for people living with HIV and knowing their HIV status (67%), on treatment (32%), and are virally suppressed (29%), is slow as HIV infections rise in the Philippines [2]. National surveillance data showed that the number of new HIV cases in the Philippines started to rise at an alarming rate during the past decade, with an increase from 311 cases identified in 2007 to 8,151 cases identified in 2016 –representing a 26-fold increase in new HIV diagnoses [3]. According to a 2014 national report, 93% of HIV cases in the Philippines were transmitted through sexual contact and were particularly concentrated among youth and young adults [4].

Despite the growing HIV epidemic in the Philippines, there have been challenges in mobilizing local and national HIV prevention, education, and testing programs [5,6]. Evidence-based public health is needed. However, a 2015 report by the World Health Organization (WHO) highlights that the body of HIV research conducted in the Philippines has been limited across all areas, including prevention, epidemiology, evaluation, and behavioral science, which are each essential to developing effective public health strategies [7, 8].

Some of the recognized key populations for HIV risk in the Philippines include men who have sex with men (MSM), transgender people, female sex workers (FSW), youth, and overseas workers [1, 6, 7]. Although the estimated number of people who inject drugs (PWID) in the Philippines has been historically low, there have been anecdotal reports suggesting a growth in this population [9]. The National AIDS and STI Prevention and Control Program for the Philippines has urged for the development of HIV prevention and public health initiatives targeted towards key populations [7]. However, an external review by the WHO of the national response acknowledged multi-level challenges in implementing HIV prevention and treatment activities, including a misalignment of healthcare priorities in the national- and city-level settings, limited healthcare infrastructure and human resources to provide prevention and treatment services to key populations, and a nascent research literature on which to build evidence-informed strategies [7].

We conducted a systematic review to examine the body of empirical literature on HIV risk and intervention programs in the Philippines. Our specific aims were to synthesize findings about population characteristics associated with HIV status or HIV risk in the Philippines, to identify and describe local HIV prevention interventions evaluated in the Philippines, and to describe the methodological characteristics of this body of research. As a secondary aim, we sought to describe differences in reported HIV risk in studies conducted before 2008 compared with studies published during or after 2008, which represents the year during which national surveillance observed noted increased HIV cases.

## Methods

**Fig 1:**
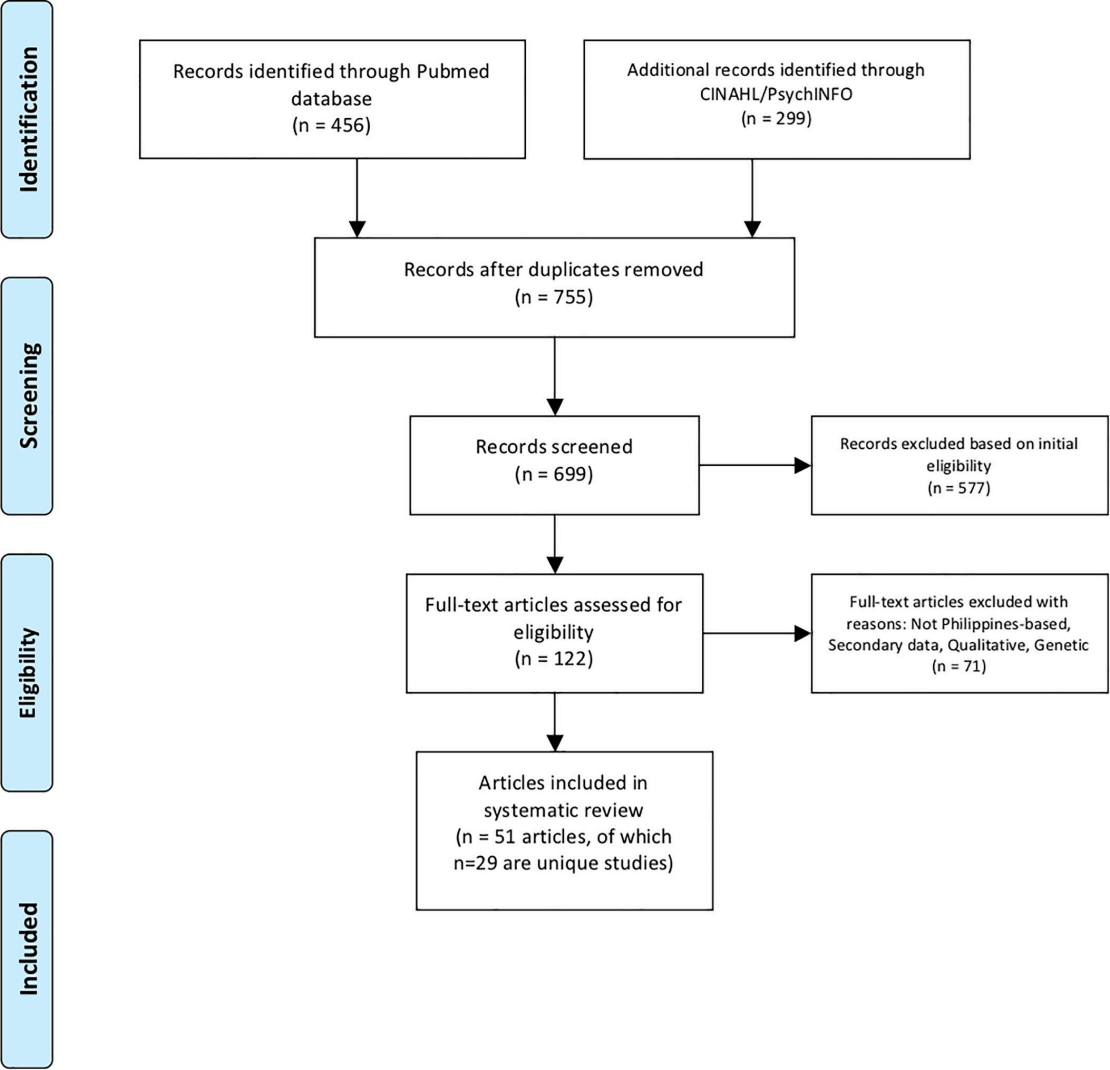
Flow chart of systematic literature review.

We conducted this systematic review in accordance with guidelines set by the Preferred Reporting Items for Systematic Reviews and Meta-Analyses (PRISMA) statement and checklist [10], which can be found in S1 Fig. Fig 1 displays the flowchart of review’s article section, inclusion and exclusion.

### Search strategy

We searched for quantitative studies assessing biological or behavioral indicators of HIV risk among Filipino/a participants in the Philippines. Studies were included if they: 1) were conducted in the Philippines; 2) sampled Filipino/a participants; 3) published in English; 4) reported quantitative findings on any of the following category of outcomes: biological risk for HIV (biomarkers/biologically confirmed HIV or other STIs); self-reported HIV status or STI diagnoses or symptoms; HIV-related sexual risk behavior (such as condomless sex, commercial sex, sex under influence of drugs/alcohol, sex with partner of HIV-positive or unknown status); injection drug use; knowledge, attitudes and beliefs relating to HIV/AIDS risk and transmission. We did not limit studies according to type of quantitative design (i.e., cross-sectional, intervention trial, etc.) or presence of a comparison or control group.

Electronic searches of PubMed, PsycINFO, and CINAHL were carried out using an a priori search strategy in April 2018. The search strategy included validated MeSH terms for HIV as well as terms related to Philippines. For example, the PubMed search used the following terms: [HIV* OR AIDS* OR HIV Infections[MeSH] OR HIV[MeSH] OR hiv[tw] OR hiv-1*[tw] OR hiv-2*[tw] OR hiv1[tw] OR hiv2[tw] OR hiv infect*[tw] OR human immunodeficiency virus[tw] OR human immunodeficiency virus[tw] OR human immuno-deficiency virus[tw] OR human immune-deficiency virus[tw] OR ((human immuno*) AND (deficiency virus[tw])) OR acquired immunodeficiency syndrome[tw] OR acquired immunodeficiency syndrome[tw] OR acquired immuno-deficiency syndrome[tw] OR acquired immune-deficiency syndrome[tw] OR ((acquired immuno*) AND (deficiency syndrome[tw])) OR “Sexually Transmitted Diseases, Viral”[MeSH:NoExp]] and Philippines. The search strings were intended to be conservative to first capture articles that relevant to the scope of the study and then identify articles meeting inclusion criteria.

This search yielded 755 records (see Fig 1 for a flowchart of the systematic review process). A team of reviewers received training in applying the inclusion criteria to research records; reviewers applied the screening criteria to an initial batch of 100 records and discussed discrepancies until reliability was achieved. Screeners were instructed to apply initial inclusion criteria liberally, such that records were retained in the search process until the team was sure that studies did not meet inclusion criteria. After excluding 56 duplicate records from the database, two reviewers screened the remaining 699 records resulting in a shortlist of 122 records that appeared to meet inclusion criteria based on information in the title or abstract. These articles were retrieved for full text review, which was performed by four reviewers (including the 2 previous reviewers) who identified 51 articles that met inclusion criteria. We excluded 71 articles because they were not based in the Philippines, were not empirical studies (i.e. editorials, commentaries, or reviews), or did not report on outcomes specified for this review. We identified several articles meeting inclusion criteria that reported different findings from the same parent research study; these articles were retained in this review.

### Data extraction and analysis

The overarching goal of this review was to summarize trends in the published literature and appraise the methodological quality of identified studies. For all research articles that met inclusion criteria, we extracted information about the year(s) of data collection, study sample and location, sampling method, study design, HIV-related outcome(s), and main findings (see Table 1). Articles that reported data from the same parent study are grouped together in Table 1, with the primary or lead article denoted by superscript “a” and subsequent articles denoted by “b”, “c”, etc.

**Table 1:** Included studies.

Study Authors	Year Collected	Sample/Location	Sampling Method	Study Design	Relevant outcome measures	Main Findings
***Study Population: General population / Other populations (i.e., female inmates, male seafarers, male drivers, male clients of sex workers)***						
***1. Agdamag et al., 2005[13]***	2002	560 female and male participants in Cebu	Did not specify	Cross-sectional	Biological tests for HIV, HCV, and HBV infections	HIV: 0/560; STD: HCV: 72/660 and HBV: 64/560
***2. Brindle et al., 1988[20]***	1985–1986	62 blood samples from Manila	Convenience sampling; multi-country study; blood samples collected from blood donors and ante-natal clinic attendees	Cross-sectional	HIV and HBV prevalence	0% HIV, 2/62 (3%) HBV posit ive results
***3. Chowdury et al., 2016[14]***	2015	2 female and 120 male Philippine seafarers	Convenience sampling from seafarer agencies	Cross-sectional	HIV knowledge/ attitudes	Low knowledge of HIV and high stigma towards persons with HIV
***4. Guevara et al., 2010[22]***	2009	100 male seafarers, ages 18–65, in Manila	Convenience sampling	Cross-sectional	HIV/STI knowledge/ attitude	STI/HIV knowledge: correct answers for transmission was average 63%, risk factors 76%, symptoms 77%, and prevention/treatment 57%
***5. Kageyama et al., 2009[24]***	2002–2007	1,590 individuals in Metro Cebu	Convenience sampling; used program surveillance data in Metro Cebu	Cross-sectional	HCV and HIV prevalence	Low HIV prevalence: 3/1,715 anti-body HIV positive samples tested, all 3 positive samples were from PWIDs. HCV prevalence: 808/1,904
***6. Murray et al., 2013[26]***	1990–2013	Patients records from clinics	Surveillance data	Cross-sectional analysis and modeling	HIV incidence/death	HIV rates developed by UNAIDS for Year 2013, Incidence: 9260 (95% CI: 4907–16,077) and Deaths: 5073 (95% CI: 3423–6397) of total population. Years 1990–2000, Incidence: 2.00 (95% CI: −15.41–13.40) and Deaths: 23.37 (95% CI: 11.46–36.25). Years: 2000–2013, Incidence: 2.32 (95% CI: −2·51–8.42) and Deaths: 1.04 (95% CI: −2.36–2.86)
***7. Reese et al., 2000[29]***	1997	318 individuals from 2 rural clinics and provincial hospital in Guimaras Province	Convenience sampling by trained nurses	Cross-sectional	Condom use behavior	Of sexually active respondents (n = 277), 12% of men had more than 1 sex partner in the last 2 months; 24% visited a CSW and among these men, 75% reported 100% condom use
***8. Saniel & De los Reyes, 2010[30]***	2008	501 male seafarers from 12 manning agencies in Metro Manila	Purposive sampling	Cross-sectional	Condom use behavior HIV knowledge and attitudes	98% heard of STIs and 60% had knowledge of HIV/AIDS. 7% of the seafarers answered 5 questions correctly on HIV transmission and prevention. 15% had commercial sex and 2% had casual sex in the last 12 months. 18% engaged in unprotected sex with their non-regular and CSW partners.
***9. Simbulan et al., 2001[31]***	Did not specify	100 female inmates in Metro Manila	Convenience sampling	Cross-sectional	HIV and STD prevalence	29/100 were positive for STD (trichomonas, gonorrhea, chlamydia, hepatitis B); 0/100 were HIV positive
***10. Velicer et al., 2009[33]***	2004–2008	396 female individuals in the Philippines	Convenience sampling; from community and academic health centers	Cross-sectional	HPV prevalence and incidence	Anogenital HPV (6/11/16/18) infection: 2.8% prevalence; anti-HPV 6/11/16/18 seropositive detected in 17.7% of participants
***11. (A) Morisky et al., 2004[54]***	2000–2005	3389 male participants (~200 from each of 18 study sites) in Southern Philippines, namely: Metro Cebu; Legaspi and Daraga; Cagayan de Oro City	CBRs with "executive officers, managers, military commanders and supervisors of each of the target populations" serving as local advisory committees.	Cross-over	STI/HIV/AIDS knowledge, attitudes and practices Condom use	Baseline vs. Post-test vs. Follow up: Condom usage (36.10% to 38.70% to 46.31%), attitudes towards condoms (21.67% to 24.55% to 25.15%) and knowledge about HIV/STI transmission (41.87% to 42.19% to 33.31%) increased significantly (p 0.01). Reported STI incidence decreased significantly (7.4% to 4.6% to 2.4%, respectively). Changes differed significantly between the intervention and control group at post-test and follow up (p 0.01).
*** (B) Morisky, Nguyen, et al., 2005[55]***	2000–2005	700 male participants (~200 taxi drivers and 150 tricycle drivers) in Lapu-Lapu and Mandawe City, Southern Philippines.	CBRs using taxi and tricycle drivers associations participation	Cross-over	HIV/AIDS knowledge, and behavior and attitude toward condom use	Repeated measures ANOVA revealed significant change from baseline to posttest and from posttest to follow-up in knowledge (F = 449.27, df = 2, p < .001), attitude (F-425.19, df = 2, p = 0.001), and behavior (F = 428.31, df = 2, p = .001) for Time × Condition, indicating differential change across time between the intervention and control group
*** (C) Regan & Morisky, 2012[56]***	2000–2005	386 male clients of FSWs from 6 regions in Southern Philippines	CBRs	Cross-over (baseline data analysis)	Condom use HIV knowledge and attitudes	For each additional year of education, the odds of men using condoms with FSW consistently increased by 13%
*** (D) Regan et al., 2013[57]***	2000–2005	2,271 male individuals from 6 regions in Southern Philippines	CBRs	Cross-over (baseline data analysis)	Condom use, substance use behavior	519/2271 use recreational drugs; Men who used drugs become sexually active earlier (OR = 1.73, 95% CI = 1.38–2.17), report 2+ recent sexual partners (OR = 2.2, 95% CI = 1.59–3.11), and report sex with a FSW (OR = 2.99, 95% CI = 2.25–4), but had a higher likelihood of using a condom during sex (OR = 1.6, 95%CI = 1.26–2.02) than non-drug users
***Study Population: Overseas Filipino Worker Candidates***						
***12. Yanase et al., 2007[36]***	2002–2004	63,249 blood donors and 69,123 overseas worker candidates in Manila	Convenience sampling; Samples from STD/AIDS Cooperative Central Laboratory (SACCL) in Manila	Cross-sectional	Anti-HIV Ab positivity; HCV prevalence	0.006% HIV prevalence in Blood donors, 0.001% HIV prevalence in OFWCs; 0.33% HCV prevalence in BDs, 0.94% HCV prevalence in OFWCs
***Study Population: Men who have Sex with Men, People who Inject Drugs, and Sex Workers***						
***13. (A) Telan et al., 2011[58]***	2007–2012	1,131 (2007) and 4,362 (2009) MSM, PWIDs, SWs in Manila. 928 (2007) and 1,241 (2009) MSM, PWIDs, SWs in Cebu. 500 (2007) and 899 (2009) MSM, PWIDs, SWs in Davao.	RDS for PWIDSs; time location sampling for MSM and SWs	Cross-sectional	HIV and HCV prevalence	Overall, increases in HIV and HCV prevalence from 2007 to 2009 among populations of MSM, PWIDs, and SW in Metro Manila, Metro Cebu and Metro Davao.
*** (B) Telan et al., 2013[59]***	2007–2012	6,045 (2011) MSM, PWIDs, and SWs in Manila. 1,162 (2011) MSM, PWIDs, and SWs in Cebu.	RDS for PWIDSs; time location sampling for MSM and SWs	Cross-sectional	HIV prevalence	Increase in HIV prevalence in 2011 in MSM, PWIDs, and SW populations in Metro Manila and Metro Cebu
***Study Population: Men who have Sex with Men***						
***14. Gangcuangco et al., 2013[21]***	2009–2010	406 MSM, ages 18 and older, in Metro Manila	Purposive sampling; CBRs from entertainment venues for MSM and from a business process outsourcing call center; van-based HIV testing	Cross-sectional	HIV diagnosis, condom use, substance use behavior Condom attitudes/beliefs	48 out of 406 (or 11.82%) MSM diagnosed as HIV-positive via rapid test (CI: 8.7–15.0). In multivariate analysis, HIV-positive status associated with working in a call center (OR = 3.37), preference for receptive anal sex (OR = 5.26), and excessive alcohol use (OR = 2.71). 46% had condomless sex during past 3 months. Only 3% used condoms consistently. Common reasons for condomless sex reported as unavailability of condoms, belief partner was HIV negative, and diminished pleasure
***Study Population: People who Inject Drugs***						
***15. Amadora-Nolasco et al., 2002[16]***	1997–1999	360 male who inject drugs in Cebu City	Purposive sampling; venues were identified before recruitment	Cross-sectional	STD symptoms HIV knowledge/ attitudes	Over three-quarters of PWIDs know three ways to prevent HIV transmission; 50/360 report STD signs and symptoms
***16. Amadora-Nolasco et al., 2001[15]***	1997–1999	360 registered FSWs and 360 freelancers FSWs in Cebu City	Purposive sampling; CBRs from specific geographic zones with temporal controls.	Cross-sectional	Condom use and STD signs HIV attitudes	Over half of FSWs know three ways to prevent HIV transmission. About three-quarters of FSWs use condoms during sexual intercourse; 54/360 registered FSW and 120/360 freelance FSW report STD signs and symptoms
***17. Verdery et al., 2017[34]***	2013	753 male PWID in Cebu and Mandaue	Respondent driven sampling	Cross-sectional	HIV prevalence, needle sharing and reuse, injection network characteristics	HIV prevalence: 52% in Cebu and 34% in Mandaue; injection at a shooting gallery: 83% in Cebu and 80% in Mandaue; sharing with other PWID: 63% in Cebu and 61% in Mandaue; inject with a used needle/syringe: 59% in Cebu and 64% in Mandaue. Higher levels of PWID network clustering in Cebu.
***Study Population: Sex Workers***						
***18. Abellanosa & Nichter, 1996 [11]***	1994–1995	160 FSWs, age 18–35, in Cebu	Multistage sampling: random and convenience CBRs from sex work venues and STD clinics	Cross-sectional	History of STD and condom use	n = 101/160 have a history of diagnosed STD; n = 101/147 report condom use > 80% of the time
***19. Amadora-Nolasco et al., 2001[15]***	1997–1999	360 registered FSWs and 360 freelancers FSWs in Cebu City	Purposive sampling; CBRs from specific geographic zones with temporal controls.	Cross-sectional	Condom use and STD signs HIV attitudes	Over half of FSWs know three ways to prevent HIV transmission. About three-quarters of FSWs use condoms during sexual intercourse; 54/360 registered FSW and 120/360 freelance FSW report STD signs and symptoms
***20. Aplasca de los Reyes et al., 2001[18]***	1996–1997	1,499 FSWs in Manila and Cebu	Purposive sampling; CBRs from clinics and street outreach.	Randomized clinical trial	Prevalence of STD (gonorrhea) and effect of STD treatment	Prevalence of N. gonorrhoeae detected among FSWs: 101/594 (16.8%) in 1994, and 120/1499 (8%) from 1996–1997. 105 FSWs were randomized to treatment (n = 77 were given 500 mg ciprofloxacin, and n = 28 were given 400 mg cefixime). Findings showed high rates of treatment failure and resistance in participants who received ciprofloxacin and adequate effects for single-dose cefixime.
***21. Hayes et al., 1990[23]***	1985–1987	25,391 FSWs from over 64 Philippines’ cities	Purposive sampling; clinics serving FSWs or in areas visited by foreigners	Cross-sectional	HIV prevalence and incidence	Low HIV prevalence: 0.8/1000 HIV positive (20 out of 25,392). Incidence rate of 2.3/1000 over one year from women that were tested twice (7 out of 2,981)
***22. Liu & So, 1996[25]***	1995	110 registered FSWs and 46 freelancer FSWs in Iloilo City	Purposive sampling; CBRs via health clinics	Cross-sectional	AIDS knowledge/ attitudes Condom use	Knowledge scores between registered and freelance FSWs not significantly different. 90–96% of SWs correctly identified HIV modes of transmission, but 25% believe they can get HIV from using public restroom. 38% of freelancers FSWs reported never using condoms vs 15% of registered FSWs
***23. Nishimura et al., 1998[27]***	1995	126 registered FSWs in Tarlac	Convenience sampling	Cross-sectional	Condom use practice Condom and HIV/AIDS attitudes/ knowledge,	90.1% reported knowing what a condom was and its application. More than 80% believe condom can prevent STDs, AIDS, and pregnancy. AIDS knowledge scores were generally high (>60%). Frequency of condom use: 38.2% always, and 19.1% never. Education status, Knowledge of condom application and effectiveness, AIDS knowledge score are all predictors of consistent vs. infrequent condom use.
***24. Urada et al., 2016[32]***	2013	86 FSWs in Quezon City	Purposive sampling; Peer outreach workers approached SWs in 3 areas of the city	Pre- and post-test	HIV Knowledge	Very low HIV transmission knowledge routes (vaginal/anal sex, breast milk, mother-child in utero, and syringe); 29% reported no HIV knowledge; at baseline, very low intentions to use condoms consistently in the next 3 months with both regular and casual partners except for street FSWs with casual partners
***25. Wi et al., 1998[35]***	Did not specify	573 registered FSWs in Manila and Cebu	Purposive sampling; from social hygiene clinic and street outreach	Cross-sectional	STI prevalence (Gonococcal and chlamydial infections)	96 (16.8%) of 570 had gonococcal infections; 109 (19.7%) of 554 had chlamydia infections; 168 (30.3%) of 554 tested had one of both infections; younger age and not registered were strongly associated with both infections
***26. (A) Morisky & Tiglao, 2010[39]***	1994–1998; and Unspecified (3 year study prior to 2004)	1,284 FSWs and 2,436 males in Legaspi, Cebu, Cagayan de Oro, and Ilo-Ilo	FSWs: EBRs via bars, night clubs, karaoke TV, and massage parlours); Male participants: CBRs including transportation (taxi cab and pedicab drivers), military, factory workers, and high-risk communities comprised the study groups.	Evaluation of longitudinal studies	Condom use behavior STI/HIV prevalence	Female participants: Over 8 months, STI prevalence = 35% infection/re-infection, 17% unknown, 48% no infection. HIV seroprevalence from 1995 to 2001 total for peer education, manager training, combined interventions, and usual care: 1/5237, 2/5672, 2/7235, and 13/6157 (respectively). Male participants: significant change on HIV knowledge from baseline to posttest to follow-up (F = 449.27, df = 2, P < .001), on condom use attitudes (F = 425.19, df = 2, P = .001), and a condom use behavior with commercial sex workers (F = 428.31, df = 2, P = .001). Condom usage (36.1% to 38.7% to 46.3%), attitudes toward condoms (21.7% to 24.6% to 25.2%), and knowledge about HIV/STI transmission (41.9% to 42.2% to 33.3%) increased significantly from baseline to posttest and 6-month follow-up, respectively (P < .01). STI incidence decreased significantly (7.4% to 4.6% to 2.4%).
*** (B) Morisky, Tiglao, Sneed, et al., 1998[40]***	1994–1998	1,394 FSWs in the Southern Philippines	EBRs via bars, night clubs, karaoke TV, and massage parlours	Cross-sectional	Condom use behavior Condom knowledge/ attitude	In general, FSWs are employed at establishments with condom use policies; 89% reported that condoms are not given or sold to sex worker's clients.
*** (C) Ang & Morisky, 2012[41]***	1994–1998	1,382 FSWs: 255 in Legaspi, 459 in Cagayan de Oro, 398 in Cebu, and 270 in Iloilo	EBRs via bars, night clubs, karaoke TV, and massage parlours	Pre- and post- test	Condom use behavior Condom use attitude	Significant intervention effects with knowledge, attitude, self-efficacy and utilization of condoms were observed between control and all intervention groups.
*** (D) Chiao et al., 2009[42]***	1994–1998	980 FSWs in Southern Philippines	EBRs via bars, night clubs, karaoke TV, and massage parlours	Pre- and post- test	HIV testing and condom use	HIV testing increased 86% from baseline (n = 980 tested) to follow-up (n = 903 tested), and was significantly associated with higher HIV/AIDS knowledge and increased condom use.
*** (E) Chiao et al., 2006[43]***	1994–1998	1,114 FSWs, aged 15–54, in southern Luzon, Cebu, Ilo-Ilo, and northern Mindanao	EBRs via bars, night clubs, karaoke TV, and massage parlours	Cross-sectional	STI prevalence diagnoses	31% of FSWs reported being diagnosed with an STI by a trained medical professional.
*** (F) Morisky, Peña, et al., 2002 [44]***	1994–1998	1,394 FBWs in the Southern Philippines	EBRs via bars, night clubs, karaoke TV, and massage parlours	Cross-sectional	Condom use with clients STI incidence	STI Incidence rate per 1000 clinical visit between 1994–1997 [and % condom use always/very often] by type of establishment: Karaoke bar = 105 [33.3] Massage parlor = 70 [26.2] Beer garden = 125 [17.3] Disco/night club = 75 [13.3] Restaurant = 252 [0.5] no establishment (e.g., freelance/brothel) = 308 [4.3]
*** (G) Morisky, Stein, et al., 2002 [45]***	1994–1998	628 FSWs in the Southern Philippines	EBRs via bars, night clubs, karaoke TV, and massage parlours	Cross-sectional	Condom use behavior AIDS knowledge	AIDS knowledge had no direct effect on condom usage, but had indirect effect through negative condom attitude.
*** (H) Morisky, Ang, et al., 2002 [46]***	1994–1998	1,383 FSWs in the Southern Philippines	EBRs via bars, night clubs, karaoke TV, and massage parlours	Cross-sectional	Condom use behavior	829/1381 reported ever using condoms. FSWs who reported consistent use condom have significantly lower rates of STI (t = 7.79, p<0.01).
*** (I) Morisky, Chiao, et al., 2005 [47]***	1994–1998	369 (baseline), 371 (post-test) FBWs	EBRs via bars, night clubs, karaoke TV, and massage parlours	Pre- and post- test	STI diagnosis in the past month Condom use behavior	Higher means of condom use in the peer education and combined intervention group compared to standard treatment group. Combined group reported approximately 60% higher mean condom use scores compared to the standard care group. Combined intervention site (Cebu) had the lowest observed STI infection rate
*** (J) Morisky et al., 2006[48]***	1994–1998	897 FBWs in the Southern Philippines	EBRs via bars, night clubs, karaoke TV, and massage parlours	Pre- and post- test	Condom use and number of STIs (self-reported)	All intervention groups (peer-only, manager-only, combined) had increased condom use and decreased number of STIs. Combined had the highest increase in condom use and largest decrease in number of STIs.
*** (K) Morisky, Chiao, et al., 2010[49]***	1994–1998	911 FSWs in Southern Philippines	EBRs via bars, night clubs, karaoke TV, and massage parlours	Cross-sectional	STI testing, condom use behavior, and substance use behaviors	Had an STI examination in the past 6 months: 1) Legaspi, peer education (85.33%), 2) Cagayan de Oro, manager training (84.65%), 3) Cebu, combined (93.78%), and 4) Ilo-ilo, control (51.06%). Consistent Condom Use (p<0.01), ORs = 1) 1.61, 2) 1.79, 3) 3.04, 4) 1.70. Daily Alcohol Consumption (p< 0.01), percentage = 1) 55%, 2) 30%, 3) 21%, 4) 45%
*** (L) Morisky, Malow, et al., 2010[50]***	1994–1998	911 FBWs in Southern Philippines	EBRs via bars, night clubs, karaoke TV, and massage parlours	Cross-sectional	HIV/STI testing, condom use behavior HIV knowledge	Received STI/HIV results in the past 6 months: 1) Legaspi, peer education 85.33%, 2) Cagayan de Oro, manager training (84.65%), 3) Cebu, combined (93.78%), and 4) Ilo-ilo, control 51.06%. Consistent condom use scale 0 never-5 always = 1) 1.61, 2) 1.79, 3) 3.04, 4) 1.70. HIV knowledge scale 0–9 = 1) 6.98, 2)6.31, 3)6.80, 4)5.90
*** (M) Sneed & Morisky, 1998[51]***	1994–1998	1,394 FSWs in Southern Philippines	EBRs via bars, night clubs, karaoke TV, and massage parlours	Cross-sectional	Condom use	Bivariate correlations between attitudes toward condoms and norms, behavioral intentions, and behavior are: -.31, -.38, -.33 respectively. Between norms, and behavioral intentions and behavior: .63 and .55. Between behavioral intentions and behavior: .72. All are significant at p < .001
*** (N) Urada et al., 2012[52]***	1994–1998	791 FSWs in Southern Philippines	EBRs via bars, night clubs, karaoke TV, and massage parlours	Cross-sectional	HIV-risk behavioral indicator, and condom use	Adolescent FSWs between ages 14–17 negotiated safer sex less with clients who refused to wear a condom than older FSWs. Both age group of FSWs engaged in the highest levels of risky behavior for STIs and inconsistent condom use
*** (O) Urada et al., 2014[53]***	1994–1998	770 FSWs in Southern Philippines	EBRs via bars, night clubs, karaoke TV, and massage parlours	Cross-sectional	Behavioral indicator of HIV risk, condom use behavior STI	28% had an STI in the last 6 months, 78% reported condom use in last sexual encounter, 36% reported being intoxicated during sex, 10% reported being high on drugs during sex
***27. (A) Urada, Morisky, Pimentel, et al., 2012[60]***	2009–2010	142 FSWs in Metro Manila	SRS and randomly sampled from 54 establishments	Cross-sectional	Condom Use	76% negotiated condom use
*** (B) Urada, Morisky, Hernandez, et al., 2012[61]***	2009–2010	143 FSWs in Metro Manila	SRS and randomly sampled from 54 establishments	Cross-sectional	Condom use behavior HIV/STI knowledge	Low HIV/STI knowledge and 58% always use condoms
*** (C) Urada et al., 2015[62]***	2009–2010	166 FSWs in Metro Manila	SRS and randomly sampled from 54 establishments	Cross-sectional	Substance use behaviors	Trafficked sex workers reported higher alcohol use and current drug use
***Study Population: Youth***						
***28. Aplasca et al., 1995[17]***	1995	845 high school students in Manila	Purposive sampling from 4 public high schools	Cluster-randomized controlled trial	Condom use behavior	11% of students (20% of males and 4% of females) reported ever having had sexual intercourse (mean age 14 years). Among these, condom use was low (24%).
***29. Balk et al., 1997[19]***	1994	10,879 youths, aged 15–24, throughout the Philippines	Nationally representative sample of youths from the Young Adult Fertility and Sexuality Study (SAFS-II)	Cross-sectional	Condom use behavior HIV knowledge	85% correctly identified one sexual mode of HIV transmission; 23% of young men reported ever using condom; among young women, 87% reported knowledge of condoms; 90% of young men reported some negative attitudes towards using condoms.
***30. Osorio et al., 2015[28]***	2007	3,044 students, ages 13–18, from 7 Philippine regions	Multistage sampling of clusters of public and private schools	Cross-sectional	Condom use behavior Condom use knowledge / attitude	44.8% used condom at first sexual initiation. 1 in 7 adolescents believed condoms are 100% effective; these adolescents were 82% more likely to have had sex than those without such belief (OR = 1.82; 95% CI 1.51 to 2.21). No association was found between risk perception and condom use.
***31. (A) Lucea et al., 2012[37]***	2005	474 young adult women, ages 15–31, in Cebu Province	Convenience sampling; interviews were a longitudinal follow up of the 1983 survey with pregnant women and their children	Cross-section	Condom use and STI history	84% reported condomless sex; 12% reported having multiple partners. 44% reported at least 1 symptom of STI. Reported STI symptoms ranged from 0–4: 56% = 0, 23% = 1, 12% = 2, 12% = 3, and 2% = 4 symptoms.
*** 3(B) Cheng et al., 2014[38]***	2005	677 male and 435 female youths, age 20–22, in Metro Cebu	Convenience sampling; interviews were a longitudinal follow up of the 1983 survey with pregnant women and their children	Cross-sectional	Substance use behaviors	risk behavior of non-same sex (NSS) behavior and same-sex (SS) behavior: Ever Smoked: 635 (67.3%) NSS and 155 (92.3%) SS Ever Drank Alcohol: 881 (93.3%) NSS and 167 (99.4%) SS Ever taken Drugs: 294 (31.1%) NSS and 101 (60.1%) SS

Note: 1) FSWs = female sex workers; FBWs = female bar workers; OFWCs = overseas Filipino worker candidates; CBRs = community-based recruitments; EBRs = establishment-based recruitments; RDS = respondent-Driven Sampling; SRS = stratified random sampling; MSM = men who have sex with men; PWIDs = people who inject drugs; SWs = sex workers; HIV/AIDS = human immunodeficiency virus/ acquired immune deficiency syndrome; STD = sexually transmitted diseases; HCV = hepatitis C virus; HBV = hepatitis B virus; 95%CI = 95% confidence interval.

2) All records with assigned letter belongs to the same dataset.

Narrative synthesis of studies was conducted based on information extracted into Table 1. Because of heterogeneity in sampling approaches, time, and indicators of HIV risk across studies, meta-analysis was not conducted. We assessed methodological characteristics of each study using a critical appraisal checklist developed by Munn and colleagues [11]. These methodological characteristics included sample representativeness, recruitment strategies, adequacy of sample size, participant drop-out or non-response, description of setting and participant, objective criteria for outcome measurements, reliability of outcome, appropriateness of statistical analysis, accounting of confounding factors, and identification of sub-populations.

Authors were not blind to any aspect of the studies. Funders of the study had no role in study design, data collection, data analysis, data interpretation, or writing of the report. The corresponding author had full access to all the data in the study and had final responsibility for the decision to submit for publication.

## Results

### Characteristics of included studies

Table 1 presents characteristics of 51 included articles from 30 independent studies. Twenty-five articles reported quantitative findings from independent studies [12–36]. Two articles reported analyses from a cross-sectional survey of sexual behaviors among adult men and women from Cebu (a large metropolitan city in the Philippines) [37–38]. Fifteen articles reported analyses from a longitudinal, quasi-experimental investigation of a community-based HIV prevention program involving female bar workers and managers in four regions of the Philippines [39–53]. Four articles reported findings from a quasi-experimental intervention to prevent HIV and STIs among heterosexuals in southern Philippines [54–57]. Two articles reported analyses of a survey of people who inject drugs in Manila, Cebu, and Davao cities [58–59]. Three articles reported analyses of a survey of FSW recruited from entertainment establishments in metro Manila [60–62].

Overall, data collection for the included studies occurred between years 1985 to 2015. Of 30 independent studies, most (n = 18) studies reported findings from data collected before 2008 and 10 studies reporting findings from data collected during or after 2008; 2 studies did not specify year of data collection, but both were published before 2008. Sample sizes per study ranged from 62 to 144,000; the latter study involved analysis of blood bank data. Female sex workers (e.g., registered and freelance) constituted the most frequently studied population (n = 11 unique studies). Fewer studies reported data on MSM (n = 3 unique studies), PWID (n = 3 unique studies), youth (n = 4 unique studies), seafarers (n = 3 unique studies), or incarcerated females (n = 1 study). One study reported on overseas worker candidates [35]. No included studies reported data on overseas workers and transgender populations, and no studies reported data on gender expression or gender identity indicators.

The majority of articles (n = 43) reported descriptive data from cross-sectional designs (including baseline data from intervention evaluations). Only 4 independent intervention studies were identified, with evaluation findings reported across 9 different articles [17,32,39,41,42,47,48,54,55].

#### HIV

Ten articles reporting data from 9 independent studies reported prevalence of HIV-positive status [13,20,21,23,24,31,34,39,58,59]. HIV prevalence across studies ranged from 0% (in 3 independent studies of 560 female and male adults in Cebu [13], 62 blood donor samples from Manila [20], and 100 incarcerated females in Manila [31]) to 52.0% in a sample of 457 participants in Cebu who were recruited using respondent driven sampling [34]. Of the 9 independent studies reporting HIV prevalence, 6 were conducted before 2008 (HIV prevalence ranging from 0.0% [12,20,31] to 0.2% [24]) and 3 were conducted after 2008 (HIV prevalence ranging from 3.3% [58] to 52.0% [34]).

#### STI

Nineteen studies reported STI diagnosis or symptoms [12,13,15,16,18,20,24,25,29,31,33,35,36,37,39,43,50,52,58]. Of those studies, four studies included Hepatitis C [13,24,36,58], three included Hepatitis B and gonorrhoeae each [13,18,20,31,35], two included chlamydia [31,35], and one for trichomonas and HPV each [31,33]. Reported prevalence ranged from a low of 0.4% in a sample of overseas Filipino worker candidates and blood donors who tested positive for Hepatitis B virus or Hepatitis C virus [36], to 63.1% history of any STI in a sample of FSW from Cebu [12]. There were no clear trends in reports of STI prevalence in studies conducted before versus after 2008.

#### Behavioral risk factors

Commonly reported HIV risk factors included self-reported condom use, and substance use behaviors. Of the included articles, thirty-one reported condom use behaviors. Assessment of condom use varied widely across studies, including measures of condomless sex with different partner types (e.g., with sex workers, multiple sexual partners, group sex, regular partners, and casual partners), frequency of condom use (e.g., using condoms always, consistently, inconsistently, and never), condom use according to type of intercourse (e.g., condom use during vaginal or anal sex), and condom use according to specific sexual events (e.g., condom use at first sex, and during last sexual encounter). Assessment of condom attitudes and knowledge also varied, with measurements including knowing that condoms prevent HIV/AIDS, pregnancy, and STI infections, as well as feeling that condoms reduce sexual pleasure or enjoyment, and that using condoms is against religion. Only five studies reported substance use outcome measures [21,34,38,57,62], and these studies varied with regard to assessment about type, frequency, and amount of substance use.

#### Intervention programs

We identified a total of four independent intervention studies aiming to prevent HIV transmission, with evaluation results reported across 9 reports [17,32,39,41,42,47,48,54,55]. One intervention study conducted in 1995 by Aplasca et al. [17] involved a cluster-randomized trial of a school-based program to improve HIV-related knowledge, attitudes, and behaviors among high school students in Manila. Findings included improved levels of knowledge about HIV biology, transmission and prevention, as well as improved attitudes and compassion for people living with HIV in the intervention versus control groups; no effects were found on intentions to engage in preventative behaviors [17]. Another intervention study conducted in 2013 by Urada et al. [32] involved a human-rights focused HIV intervention for sex workers in Manila. Participants completed a single 4-hour intervention providing HIV and STI knowledge and prevention strategies, and contextualized risk and protective factors in accordance with the laws, systems, and social milieu regarding sex work, violence, and discrimination. In pre-post analysis, participants reported higher levels of knowledge about HIV reproductive health, human rights, research ethics, and intentions to receive an HIV test [32].

Two intervention programs were quasi-experimental studies. First, a 3-year study conducted from 1994–1998 by Morisky et al. [39] used community-based participatory methods to train managers and peer educators on HIV and STI prevention at 130 entertainment establishments in 4 regions in the Philippines. Trained managers and peers then implemented and disseminated information within their establishments. In post-test evaluation analyses, FSW employed in these establishments reported significant improvements in consistent condom use, improvements in HIV testing, and reductions in STI infections [39,42,47,48]. Additional analyses showed that improvements in condom use were strongest in establishments that also instituted condom use policies for employees and patrons [41]. Second, a 3-year quasi-experimental study conducted from 2000–2005 by Morisky et al. [54] used community-based participatory methods to provide HIV prevention education training to peer leaders recruited from six male populations in the southern Philippines: military members, police and firemen, construction workers, taxi drivers, pedicab drivers, and community residents; trainees were then expected to educate 10 or more peer network members on HIV and STI prevention. In post-test evaluation analyses, intervention participants reported lower levels of STI infections as well as improvements in HIV and STI knowledge, attitudes toward condoms, condom use behavior, discussion about HIV with co-workers, and exposure to HIV prevention education, compared with those in the control [54,55].

Finally, another prospective STI treatment study was identified, but was not considered a scalable HIV prevention intervention compared with the four studies described here. In this STI treatment study conducted in 1996–1997 by Aplasca de los Reyes et al. [18], FSWs from Manila and Cebu testing positive for *N*. *gonorrhoeae* were randomly assigned to receive a single dose of ciprofloxacin (500 mg) or a single dose of cefixime (400 mg), and were re-evaluated 4–7 days later. Findings showed high rates of treatment failure and resistance in participants who received ciprofloxacin and adequate effects for single-dose cefixime [18].

#### Methodological appraisal of the included studies

For selecting participants, most of the study participants were recruited using sampling methods based on non-randomized sampling strategies, including convenience and purposive sampling such as venue-based (e.g., community or establishment) recruitment approaches or from surveillance studies. Common examples of venue-based recruitments were via bars, nightclubs, karaoke TV lounges, massage parlors, and health clinics. One study reported data from a nationally representative youth survey [19], and two studies reported data from respondent driving sampling surveys [34,58]. Although all studies indicated the type of recruitment strategy used (e.g., convenience sampling, etc.), almost 13 did not specifically detail about how participants were recruited. All studies had adequate sample size to perform analysis, and had high participant response rate. Most studies (n = 26) provided adequate description of their participant samples and the study settings.

When considering outcomes, nineteen studies did not use objective standard criteria for measuring outcomes (i.e., validity) such as a biologically-confirmed results. Self-reported outcomes were primarily assessed and there were noticeable inconsistent measurements in condom use and substance use behavioral measures and HIV-related knowledge and attitude measures. Statistical analyses for each study were conducted appropriately based on study design. Of the four intervention studies, one used a cluster randomized control trial design, two used non-randomized comparison groups, and one used a pre-post evaluation design. Intervention studies assessed self-report behavioral outcomes only; none measured biological or objective indictors of HIV risk.

## Discussion

Despite the rise in HIV infections documented in the Philippines’ national surveillance reports since 2008, this review highlights the limited body of published research on HIV infection and risk factors in key populations, a paucity of research on interventions to promote HIV prevention and testing in the Philippines, and opportunities for improving methodological rigor in future research. Overall, we identified 51 published quantitative papers reporting on HIV- or STI-related biological, behavioral, or social-cognitive findings from 30 unique studies conducted in the Philippines. The majority of papers identified in this review reported on data collected before 2008; only 10 papers (reporting on 7 discrete studies) reported data collected during or after 2008. Moreover, this review identified only 4 HIV prevention intervention evaluations in the published literature.

Prior to 2008, FSWs constituted the population most frequently studied in HIV research conducted in the Philippines. After 2008, a small number of studies included MSM and PWID. Indeed, the papers by Gangcuangco and colleagues [21] and Telan and colleagues [59,60] and are among the only identified published studies which recruited and reported specifically on HIV risk in MSM populations. Findings from PWID were included in four reports–one involving surveillance data from an earlier phase of the epidemic [16], three conducted after 2008 by Verdery and colleagues [34] and by Telan and colleagues [58,59]. Four studies included youth; two were conducted before 2008 [17, 19] and two after [28, 38]. No identified studies specifically reported on overseas workers and transgender populations.

This review identifies a need to improve the body of knowledge about HIV risk and transmission among key populations in the Philippines. Research targeting MSM, PWID, and transgender populations is needed to understand the transmission risk factors and specific structural, social, behavioral, and epidemiological factors impacting these groups. While there is a considerable body of HIV prevention intervention research focusing on MSM and PWIDs in other parts of the world [63,64], none have been specific to the Philippines. It remains unclear whether existing prevention interventions are adaptable or require distinct design for this national context. Additionally, despite an estimated global HIV prevalence of 19% [65], and anecdotal reports that suggests a growing burden of HIV among transgender women in the Philippines [66], none of the included studies have focused on or included this key population. It is possible that researchers in the Philippines have aggregated transgender populations within the MSM rubric [67]. Given HIV epidemiological trends within the Philippines and evidence from other settings about the disproportionate prevalence of HIV among transgender people, future research must disaggregate transgender and MSM populations and resist the conflation of gender and sexual identities [68]. Additionally, given that UNAIDS surveillance data points to youth and young adults being affected [2], more biological and behavioral research data and interventions are necessary to understand the epidemic among youth and young adults in the Philippines, especially those who are also members of MSM, PWID, or transgender populations.

Most of the studies identified in this review used cross-sectional surveys with convenience samples, involving mostly descriptive measures, which limit the generalizability of the research. While these study designs are useful in exploratory investigations, it is imperative for researchers to increase the rigor of investigations by using longitudinal and experimental studies in order to examine more complex research hypotheses (e.g., testing hypotheses about social-behavioral determinants of HIV infection) and to test interventions [68].

Moreover, as the majority of includes studies were conducted prior 2008, researchers must also examine biomedical factors that might determine or mitigate the growth of HIV in the Philippines–e.g., factors associated with medication adherence and viral load suppression among PLHIV (i.e., treatment-as-prevention), access to and use of biomedical prevention such as post-exposure prophylaxis and pre-exposure prophylaxis, home-based testing, and male-circumcision. Given the political climate regarding drug use in the Philippines, the viability of harm reduction and needle exchange programs for HIV prevention must be carefully considered [69,70].

The literature identified in this review was also noteworthy with regard to the scarcity of investigation into psychosocial and ecological factors that contribute to HIV risk, mental health, personal and community empowerment, stigma, and substance use. While it is important to continue assessing condom use-related measures in key populations, it is imperative that researchers examine contextual conditions and co-morbid health factors associated with low condom use as well as the broader social-structural drivers of HIV risk and infection, in order to understand factors affecting the acceptability and feasibility of bio-behavioral preventative strategies.

Taken together, these findings provide initial insight into the increasing and shifting HIV epidemic in the Philippines, from an initial concentration among FSWs to including populations such as MSM and PWIDs. These findings also suggest the need to develop social-contextual frameworks to prioritize HIV prevention strategies and contextualize HIV risk/prevention according to the lived experiences of key populations. Assessment of clinical and service provider capacities in responding to HIV risk and infections is also important to guide research and build a stronger public health infrastructure.

Limitations of this review must be considered. First, although this review followed a systematic search protocol and a priori inclusion criteria, it is possible that not all relevant articles were identified. Second, meta-analysis was not deemed appropriate due to heterogeneity in samples, methods, measures, and time of data collection. Third, the review included only English-language publications. Fourth, the generalizability of the review might be limited given the specificity of the context for this review and the ongoing evolution of the Philippines’ national epidemic.

This is the first known systematic review to provide evidence about and identify gaps in published research about risk groups, risk factors, and intervention approaches addressing the Philippine’s HIV epidemic. Studies revealed a nascent body of literature, especially with regard to intervention research, biomedical prevention, and key populations currently impacted by the HIV epidemic such as PWIDs, MSM, and transgender populations. Future HIV research studies in the Philippines must use rigorous research methodologies including purposeful sampling strategies and validated measures (including biological assessments for HIV and STIs, and established measures for substance use, condom use, and mental health). Furthermore, given the evolving epidemic in the Philippines, researchers should capitalize on opportunities to implement and evaluate bio-behavioral intervention strategies including pre-exposure prophylaxis, treatment-as-prevention, and regular HIV testing with key populations. Use of online data recruitment and data collection approaches can improve access to hard-to-reach or remote populations; online research might be especially useful for reaching members of historically stigmatized groups. A multi-disciplinary research agenda for understanding and addressing HIV transmission in the Philippines must span across various domains of research including prevention, epidemiology, treatment, and behavioral and social science.

## Supporting information

S1 Fig PRISMA checklist (2009). (DOC)
